# Determination of Air Enthalpy Based on Meteorological Data as an Indicator for Heat Stress Assessment in Occupational Outdoor Environments, a Field Study in IRAN

**Published:** 2016-08-21

**Authors:** Hamidreza Heidari, Farideh Golbabaei, Aliakbar Shamsipour, Abbas Rahimi forushani, Abbasali Gaeini

**Affiliations:** ^a^ Research Center for Environmental Pollutants, Qom University of Medical Sciences, Qom, Iran; ^b^ Department of Occupational Health, School of Public Health, Tehran University of Medical Sciences, Tehran, Iran; ^c^ Department of Physical Geography, School of Geography, University of Tehran, Tehran, Iran; ^d^ Department of Epidemiology and Biostatistics, School of Public Health, Tehran University of Medical Sciences, Tehran, Iran; ^e^ Department of Sport Physiology, School of Physical Education and Sport Science,, University of Tehran, Tehran, Iran

**Keywords:** Enthalpy, Heat Stress, Climate, Environment, Index

## Abstract

**Background:** Heat stress evaluation and timely notification, especially using meteorological data is an important issue attracted attention in recent years. Therefore, this study aimed at answering the following research questions: 1) can enthalpy as a common environmental parameter reported by meteorological agencies be applied accurately for evaluation of thermal condition of outdoor settings, and 2) if so, what is it’s the best criterion to detect areas in stress or stress-free situations, separately.

**Methods:** Nine climatic regions were selected throughout Iran covering a wide variety of climatic conditions like those, which exist around the world. Three types of parameters including measured (ta, RH, Pa and WBGT), estimated (metabolic rate and cloth thermal insulation), and calculated parameters (enthalpy and effective WBGT) were recorded for 1452 different situations. Enthalpy as a new indicator in this research was compared to WBGT in selected regions.

**Results:** Altogether, a good consistency was obtained between enthalpy and WBGT in selected regions (Kappa value: 0.815). Based on the good ROC curve obtained using MedCal software, the criterion of the values more than 74.24 for the new index was determined to explain heat stress situation for outdoor environments.

**Conclusions:** Because of simplicity in measurement, applicability of the indicator for weather agencies, the consistency observed between enthalpy and a valid as well as accurate index (WBGT), sensor requirements which take only a few seconds to reach equilibrium and so on, enthalpy indicator can be introduced and applied as a good substitute for WBGT for outdoor settings.

## Introduction

Heat stress as a physically harmful agent especially for the outdoor environment occupants has been always posed as a concerned issue for the corresponding governmental organizations related to health and safety of people. Outdoor workers such as construction workers, agricultural workers, asphalt and road construction workers etc., who spend more than one- third of their time outside, are one of the most important groups who can be influenced by the adverse effects of the heat. In fact, in addition to environmental parameters, personal parameters including work load or metabolism as well as thermal insulation of the clothes associated with the work can probably produce additional body heat load for the last group.

High temperature especially when combined with high relative humidity persists for several days (heat waves), and if night time temperatures do not drop, extreme heat can be mortal. Impacts will likely vary by region, the sensitivity of populations, the extent and length of exposure to heat as well as workers' ability to adapt to heat^1^. It can also be aggravated by considering global warming and climate change which is increasingly reported recently all over the world. The average warmer temperatures will likely lead to hotter days and more frequent and longer heat waves. This, in turn, could increase the number of heat-related illnesses and deaths^2^. The effects of rising temperature as a result of climate change from point of view of occupational heat stress unfortunately has not been considered as commonly as other related issues such as ecological and environmental impacts^3^. Therefore, paying attention to ways of identifying health impacts arising from heat stress as a result of climate change in order to adopt preventive and controlling measures is deemed very important. In this respect, assessment of the thermal situation of exposed persons using meteorological data, reported daily for each weather station, instead of using many thermal indices related to other environmental and personal parameters associated usually with some estimation, advanced sensors or sophisticated computations, can be a good idea. As such, the thermal condition at any time can be determined or even predicted simply and timely for the notification of exposed persons in order to take protective measures.

A lot of environmental variables have been combined to create thermal indices for evaluation of thermal stress or comfort from previous decades till now (more than 45 indices from 1905 to 2005)^4-6^. Wet bulb globe temperature (WBGT) is a valid and important heat index currently used all over the world. This index adopted by ISO-7243 (1989) is calculated by three parameters including dry air temperature, natural wet temperature and glob temperature for outdoor environments^7^.

In spite of the simplicity and wide applicability of WBGT, as well as the possibility to measure heat stress both for indoor and outdoor settings, due to the dependency of the index to the global temperature, and the need for some estimations (such as metabolic rate and thermal insulation of clothes), the calculation of the index by the data of Meteorological Organization still remains impossible and so, the applicability of the index for public awareness about thermal status of the environment through the Meteorological Organization has encountered a restriction. On the other hand, due to required accuracy and availability, the need for calibrated equipment and qualified personnel, highly sophisticated calculations, for example in the case of Predicted Heat Strain or PHS, and the need for estimation of some parameters, as well as some intrinsic limitations associated with them, the use of many of thermal indices has faced with restrictions^5,8^.

Many attempts have been made so far to assess and report the thermal stress condition using directly measured meteorological parameters. In Australia, south Asia, South Africa, south and Central America during 1975-2000, WBGT was estimated using environmental parameters recorded in meteorological stations, which increased from 0.5 to 1 degree of centigrade because of global warming^9^. A similar investigation was on physical models to predict WBGT using meteorological data^10^. Environmental stress index, ESI, is another index resulting from combination of three directly-measured meteorological parameters including air temperature, humidity and solar radiation, which is calculated as follows^11^:

ESI=0.62.t_a_-0.007 .RH+0.0002 .SR+0.0043 .(t_a_.RH)-0.08(0.1+SR)^-1^ (Eq. 1)

This index introduced as a substitute for current and valid heat stress index, WBGT, and demonstrated good correlation with it, has at least three advantages related to WBGT: 1) Measurement of solar radiation and relative humidity instead of globe bulb temperature and natural wet temperature, respectively. 2) Applicability for the meteorological organization to report environmental heat stress condition from directly measured meteorological data. 3) The need for less stabilizing time for the sensors to reach equilibrium with the environment. However, solar radiation was not a common parameter in many synoptical stations (for example in Iran) and so, the calculation of the index is impossible.

The area coverage of different climate types in Iran- which is 35.5% hyper-arid, 29.2% arid, 20.1% semi-arid, 5% Mediterranean and 10% wet (of the cold mountainous type) -has caused more than 82% of Iran's territory to be located in the arid and semi-arid zone of the world. Diversity of the Iran's climates on the one hand and the large number of workers who work outdoors, on the other hand, indicate that heat stress as a major health risk can be faced by a lot of workers all around the country and strike them especially in hot months (from May to October). Therefore, the selection or development of an appropriate heat stress or comfort index can be helpful in the prevention of and protection from heat related disorders and illnesses.

According to the aims of the study, indicators should be used not only to represent truly thermal situation of the outdoor environments, but also to calculate it simply using meteorological data reported daily by each synoptical station. Since air enthalpy as a common environmental parameter currently calculated and reported simply by meteorological data, is a measure of the heat content of substances, and can be used conveniently for finding the amount of heat necessary for certain processes, it is, therefore, suitable for predicting the condition of air in a thermal space^12^. This variable is often used as a comfort indicator for installations and cooling systems, indicating a quantity of thermal energy to be removed from the environment to enable thermal conditions of survival inside an installation^13^.

This study aimed at answering the following research questions: 1) can enthalpy as a common environmental parameter reported by meteorological agencies be applied accurately for evaluation of thermal condition of outdoor settings, and 2) if so, what is its best criterion to detect areas in stress or stress-free situations, separately.

## Methods

### Measurement of environmental parameters

Basic parameters necessary for calculation of enthalpy including ta, RH and Pa were measured from March to October (six months during which the weather is nearly warm to hot in many parts of Iran) at 9:00 am, 12:00 am and 15:00, as representing early, middle and end of the work shift, respectively. For measuring data in a way which would cover a wide range of environmental parameters, 9 regions of Iran with different climates were selected (Table 1). All the selected climatic regions usually experience moderate to hot temperatures with different intensity of humidity in spring and summer. Like other required environmental parameters, WBGT index is also measured simultaneously in each station using an advanced calibrated Heat Stress Monitor (Casella Microtherm WBGT, UK). This index can be also calculated for outdoor environments with solar radiation as follow:

**Table 1:** Description of studied climate categories

**Climate** **category**	**Nominal category** ^a^	**Synoptic stations**
Site 1	Arid, cool and warm to very warm region	Tehran, Semnan, Qazvin, Arak, Qom
Site 2	Semi-arid, moderate and very warm region	Ilam, Ahvaz
Site 3	Semi-arid, cool and warm region	Bojnourd, Zanjan, Shiraz, Mashhad
Site 4	Arid, cool and warm region	Birjand, Kerman
Site 5	Arid, cool and very warm region	Esfahan, Zahedan, Yazd
Site 6	Arid, moderate and very warm region	Bushehr, Chabhar, Bandarabbas
Site 7	Humid, cool and warm region	Noshahr, Ramsar, Sari
Site 8	Semi Humid, cool and warm region	Gorgan, Yasouj, Ghasre-Shirin
Site 9	Post Humid, cool and warm region	Bandar Anzali, rasht

^a^ Cool in the nominal category is one of the properties of winter weather conditions, not necessarily spring and summer weather conditions which is emphasized on in this study (source: Heidari et al, 2015).

WBGT=0.7t_nw_ +0.2t_g_+0.1t_a_ (Eq. 2)

Where is, t_nw_: natural wet temperature (°C), t_g_: globe bulb temperature (°C), t_a_: air temperature (°C). A digital manometer (Lotron PHB 318, Taiwan) was used for recording of relative humidity and barometric pressure in this study. Overall, 242 outdoor worker conditions from nine selected regions were monitored. On the whole, 1452 measurements were recorded for each parameter in the selected areas (9 climatic regions multiplied by 242 station multiplied by 3 repetitions during shift work and also twice during the study including spring and summer). Calculation of the enthalpy was performed using enthalpy’s general equation of moist air^13^(Eq. 3).

(Eq. 3) $$h=1.006.t+\frac{RH}{p_{B}}.10^{\left(\frac{7.5.t}{237.3+t}\right)}.(71.28+0.0052.t)$$

where, ta: air temperature (°C), RH: relative humidity (%), P_B_: barometric pressure (mm.Hg). The unit of the enthalpy is kJ/kg of dry air and pressure is mmHg.

### Determination of a cut of point for Enthalpy

In order to test the applicability of enthalpy as an indicator of heat stress evaluation, at first, the consistency between enthalpy and WBGT was determined. If a good agreement was observed between the two indicators, then, determination of a cut- off point for enthalpy values, which in stress and stress-free conditions can be truly determined, was required. Therefore, in the second stage of the study, thermal condition of each situation was defined as two groups including 1) stress status and 2) safe or stress-free status. For this case, measured and standard WBGT for each station were compared. To compare the measured and standard WBGT amounts or reference values, the effects of thermal insulation of the usual clothes worn by workers in the hot months were first estimated according to ISO-9920 (2007)^14^ and then correction was made for simultaneously-measured WBGT, if necessary. This value is called "effective WBGT" during the study^15^.This amount was then compared with reference values adopted by ISO-7243 (1989)^16^. All the workers were supposed to have been acclimatized and the metabolic rate of the common works in each station ranged from moderate to heavy according to ISO- 8996 (2004)^17^. Finally, the enthalpy criterion was determined based on these two determined status and using a receiver operating characteristic (ROC) curve obtained by MedCal software, version 8.0.

### Statistical analysis and Roc curve

As mentioned above, to determine an appropriate enthalpy's criterion for evaluation of outdoor thermal environment, WBGT as the gold standard was divided into two groups (stress and stress-free condition). Then enthalpy was considered to be independent variable and WBGT the classification (dependent) variable. Based on obtained area under the ROC curve, as well as sensitivity and specificity at 95% confidence interval, the enthalpy criterion capable of predicting safe or stressed situations in outdoor environment with a good approximation can be obtained. Finally the consistency of two indicators, WBGT and enthalpy, was examined using Kappa coefficient test.

## Results

The results of mean and standard deviation of three groups of parameters including measured, estimated and calculated parameter in 9 climatic regions of study are shown distinctly in Table 2. The wide range amount of each environmental parameter in this research was observed so that, the range of t_a_, RH and P_a_ was 14.6 to 46 °C, 20.9 to 93.8% and 594.9 to 775.6 mmHg, respectively. In such a condition, the calculated enthalpy values were between 39.41 to 113.27 kJ.Kg^-1^ of dry air. Furthermore, total means and standard deviations of estimated thermal insulation of clothes (0.82±0.18 clo) and metabolic rate of workers (181.28±23.05W.m-^2^) revealed that as expected measured WBGT and effective WBGT have not significant differences and the common workloads in outdoor settings which were studied ranged from moderate to heavy.

**Table 2:** Measured, estimated and calculated parameters in this study for each climatic region (n=1452)

**Climatic category**	**Parameters (M ± SD)** ^a^							
	**Measured**				**Estimated**		**calculated**	
	**ta (°C)**	**RH (%)**	**P**_a_ ** (mmHg)**	**WBGT**_meas_ **(°C)**	**I**_cl_ ** (Clo)**	**M (W.m-2)**	**h (KJ.Kg** ^-1^ **)**	**WBGT**_effc_ ** (°C)**
**Site 1 (**n=150)	30.89 ±6.87	40.82 ±15.05	658.49±7.13	23.93 ±3.70	0.86 ±0.23	164.60 ±23.17	61.54 ±9.37	23.24 ±3.51
**Site 2 (**n=162)	35.34 ±6.17	43.62 ±12.89	753.08 ±3.45	28.10 ±3.55	0.75 ±0.12	157.52 ±22.32	74.36 ±11.01	28.69 ±3.34
**Site 3(**n=180)	32.17 ±3.97	43.47 ±7.54	668.47 ±1.17	25.90 ±2.81	0.91 ±0.18	180.33 ±11.53	70.41 ± 8.90	25.18 ±2.65
**Site 4 (**n=180)	28.48± 4.94	40.46 ±8.82	607.71±10.65	22.14 ±3.18	0.82 ±0.20	182.16 ±28.72	59.13 ±8.55	22.56 ±2.77
**Site 5 (**n=144)	37.32 ±3.50	33.58±5.62	651.94 ±7.07	28.78 ±2.77	0.76 ±0.11	186.04 ±20.01	77.90 ±10.63	28.88 ±2.83
**Site 6 (**n=162)	36.93 ±3.00	61.19±8.88	753.60 ±1.86	32.73 ±2.11	0.69 ±0.06	192.50 ±18.71	99.62 ±9.10	32.09 ±2.90
**Site 7 (**n=144)	25.38 ±6.59	70.36 ±11.22	761.07 ±3.08	22.92 ±5.47	0.74 ±0.04	180.83 ±15.84	62.97 ±16.97	22.94 ±4.88
**Site 8 (**n=150)	30.53 ±4.05	59.14 ±10.30	753.60 ±4.95	26.70 ±3.73	0.82 ±0.23	184.80 ±28.69	74.25 ±15.84	26.97 ±3.37
**Site 9 (**n=180)	28.10 ±3.15	72.97 ±8.11	755.60 ±5.79	25.83 ±2.51	0.77 ±0.11	186.08 ±14.50	73.00 ±8.48	25.38 ±2.60
**Total (**n=1452)	31.63 ±6.18	51.78 ±16.86	705.98 ±57.19	26.30 ±4.57	0.82 ±0.18	181.28 ±23.05	72.52 ±15.95	26.52 ±4.23

^a^ t_a_: dry air temperature (°C), RH: relative humidity (%), WBGT_meas_: measured WBGT (°C), I_cl_: dry thermal insulation (clo), M: metabolic rate (W.m^-2^), h: enthalpy (kJ.Kg^-1^ of dry air), and WBGT_effc_: effective WBGT (°C).

**Figure 1:**
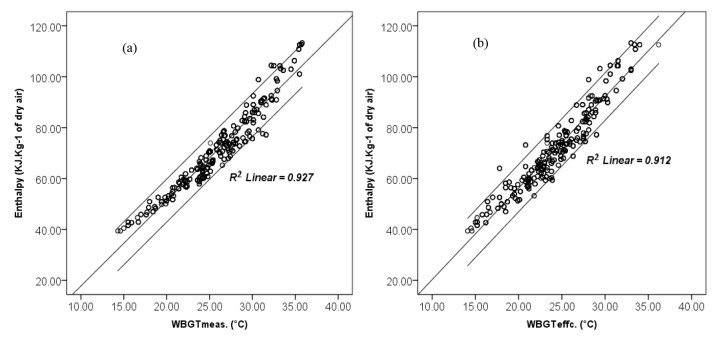
Correlation of enthalpy with measured (a) and effective WBGT (b) (regardless of climatic categorization)

**Figure 2:**
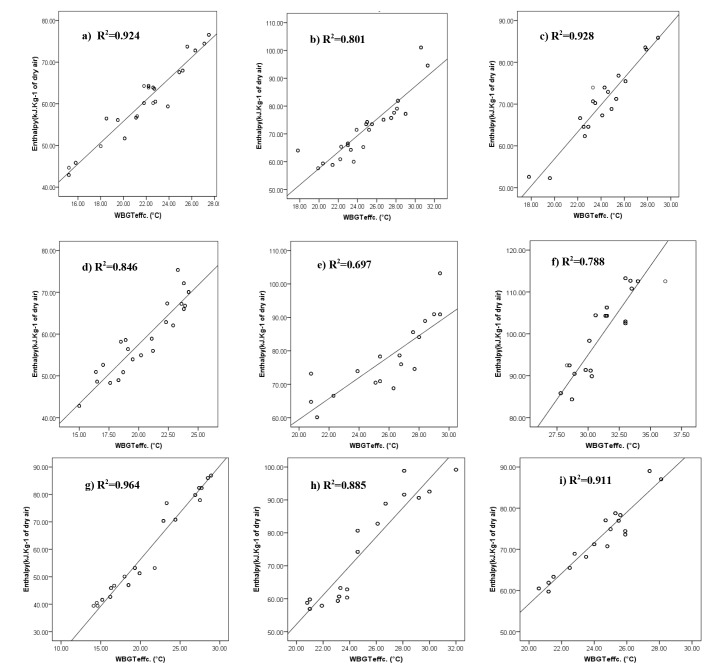
Correlation of effective WBGT and enthalpy in different climatic conditions; (a) refer to site 1; (b) refer to site 2; (c) refer to site 3; (d) refer to site 4; (e) refer to site 5; (f) refer to site 6; (g) refer to site 7; (h) refer to site 8; and (i) refer to site 9. (See Table 1 for nominal categorization of each site)

The correlation of both measured WBGT and calculated effective WBGT, with enthalpy in different conditions but concurrently were evaluated. The obtained results regardless of climatic categorization in this study as presented in Figure 1, showed good linear correlation coefficient for both comparisons (r> 0.95). Besides, comparisons were done by considering climatic categories and correlation of enthalpy with effective WBGT examined in different sites, separately (Figure 2).

**Figure 3:**
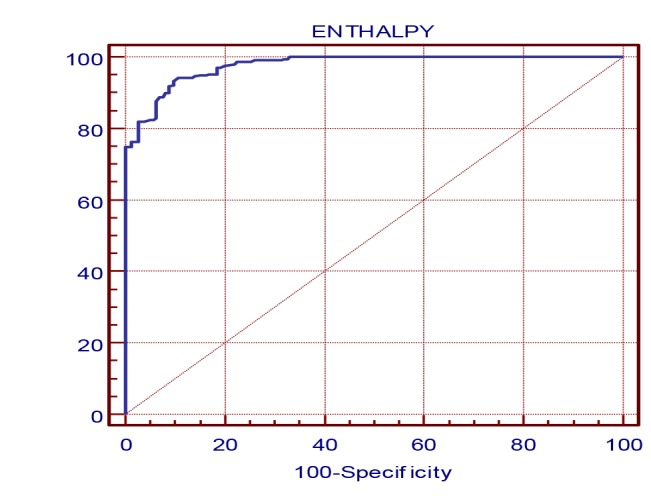
Obtained ROC curve for the enthalpy based on WBGT_effc_

As shown in Figures 1 and 2, all in all not only a correlation can be seen between WBGT (both measured and effective) and enthalpy, but also appropriate determination coefficients were obtained between enthalpy and effective WBGT for each studied climate in this research. Therefore, enthalpy criterion was determined based on WBGT_effc._ as a gold standard. The selection of the WBGT_effc._ was due to considering the thermal insulation of the clothes. At confidence interval of 95% (95% CI), with a very good sensitivity and specificity, the values more than 74.24 were selected as a criterion of enthalpy to assess thermal situation of outdoor environments (Table 3 and Figure 3).

**Table 3:** Enthalpy criterion from MedCal software (Based on WBGTeffc)

** Independent**	**variable Enthalpy (KJ.Kg-1 of dry air)**
Classification variable	WBGTeffc.(°C)
Positive group (n)	551
Negative group (n)	901
Area under the ROC curve	0.977
Standard error	0.005
95% Confidence interval	0.968 to 0.984
Sensitivity (95% CI)	94.2 ( 91.9, 96.0)
Specificity (95% CI)	89.3 ( 87.1, 91.3)
Criterion	> 74.24

## Discussion

This study aimed to assess the capability of the enthalpy as a simple environmental parameter reported daily by meteorological agencies for evaluating thermal conditions in different climates. The climatic regions selected in this study have covered wide ranges of environmental parameters which have in turn, resulted in arid, semi- arid, humid and post humid areas with different intensities of air temperature ranging from moderate to very warm conditions (Table 1). This diversity of the climates makes it possible that the results be used in any part of the world with similar climates. The results in Table 2 demonstrate that site 4 and then site 7 have minimum amounts of WBGT (22.56± 2.77 °C and 22.94± 4.88 °C, respectively) related to humid, cool and warm regions and arid, cool and warm regions, respectively in this study. On the other hand, in the cases of site 6 and then 5, maximum amounts of WBGT were recorded (32.09±2.90 °C and 28.88± 2.83 °C, respectively) (Table 1 and 2). These latter areas located in arid, moderate and very warm regions and arid, cool and very warm regions, belong to southern and central regions of the country and usually suffer from heat effects in all the spring and summer seasons. Similar results can be seen likewise in some investigations conducted on heat stress or comfort, tourism and climatic change issues in Iran,^18-20^. The mean and standard deviations of thermal insulation of clothes worn by workers in this study were estimated to be less than 1 clo, so there were no significant differences between measured and effective WBGT (*P*< 0.001).

According to Eq. 2, the most important contributing parameter in computation of WBGT is natural wet temperature (t_nw_) indirectly measured instead of relative humidity. Comparing relative humidity, t_nw_ is influenced by vapor pressure and needs more consideration as well as more sensor equilibrium time when it is going to be measured^21^. This is while enthalpy is calculated according to Eq.3 using directly measured parameters. This finding can be comparable with the study of Moran et al., (2003), in which, they introduced ESI as an alternative for WBGT for outdoor environments ^11^. They also stated that substitutions of directly measured parameters including relative humidity (RH) and solar radiation (SR) for the ESI computation with indirectly measured parameters, the natural wet temperature and globe temperature, respectively, can be considered as an element of strength of an index^11^. Therefore, the obtained results based on enthalpy are probably more realistic than WBGT. Monitoring thermal condition using WBGT is accompanied by an overestimation^22-25^. For example, in a study in which warm and humid conditions of tropical agricultural environments were simulated, appropriateness of WBGT for monitoring such conditions, which is similar to, conditions of more than 70% of developing countries were investigated. The results showed WBGT was a conservative index for monitoring these conditions^23^. Contrarily, in other studies WBGT introduced as a valid and simple heat stress index. For example, in an investigation on heavy activities in dry and hot climates of Iran, WBGT was chosen as optimal heat stress index due to a lot of limitations associated with other thermal indices^26^.This may be duo to selection of indices in that study. They compared WBGT, Discomfort Index (DI), Effective Temperature (ET), Corrected Effective Temperature (CET) and Heat Stress Index (HIS), which most of them, except HIS, are used often as an indicator of thermal comfort. For example, ET and CET cannot reflect all the important factors of outdoor heat stress since it is designed mainly for the indoor condition ^27^.As presented in Table 2, the amounts of WBGT have exceeded threshold limit value recommended for moderate work (28 °C)^16^ in regions 2, 5 and 6 (28.69± 3.34 °C, 28.88± 2.83 °C and 32.09± 2.90 °C, respectively). These areas were similarly evaluated using enthalpy indicator. The criterion of enthalpy was determined to be equal to 74.24 KJ.kg^-1^ in Table 3; i.e. if enthalpy calculated using three mentioned environmental parameters (ta, RH and Pa) was more than 74.24 in terms of KJ.Kg^-1^ of dry air, the thermal situation of the outdoor setting is probably in stress condition and when equal to or less than this value, the thermal condition is probably safe or stress-free.

Comparison of WBGT and enthalpy with and without considering climatic categorization (Figures 1 and 2) showed good correlation coefficient for both conditions. The least correlation coefficient was observed for regions with very high temperature and very low humidity condition (site 5 in Table 1), as well as for regions with both very high temperature and very high humidity condition (site 6 in Table 1 and Figure 2). Probably enthalpy indicator is not the most appropriate choice for thermal evaluation of outdoor environment in such conditions. Similarly, this is a limitation for using WBGT in climates with very hot and humid conditions. The applicability of the WBGT index will also be restricted in conditions with very low air velocities or stagnant air ^8^.

Table 3 and Figure 3 showed that based on excellent sensitivity (95% CI), 94.2 (91.9, 96.0), and specificity (95% CI), 89.3 (87.1, 91.3), obtained for enthalpy as an independent variable against WBGT_effc._ (as classification variable), as well as the area under the ROC curve (0.977), the criterion of the values more than 74.24 can probably explain heat stress situation for outdoor environments. Since this value can be applied without any need for estimation or correction of personal parameters affecting thermal comfort, as well as good consistency observed between enthalpy and WBGT, it can be used as a substitute for WBGT index. Moreover, in contrast to WBGT which relies on measuring natural wet temperature and globe blue temperature (both of them are not common parameters reported by every weather station)^28^, enthalpy can be calculated and reported simply using common meteorological data.

Integration of dry bulb temperature and relative air humidity is important to assess thermal comfort and heat stress in certain conditions^29^. These variables which considered both of them in enthalpy are responsible for quantifying the capacity of thermal energy in the environment^30^.

Finally, based on comparisons made between WBGT and enthalpy indicator presented in this study, and according to obtained Kappa value, the total consistency of two indices was good (Kappa value = 0.815). Likewise, as Table 4 shows, except site 9 (regions with post humid, cool and warm weather conditions) the Kappa values have been good in almost all other regions. On the other words, in conditions such as cite 9, thermal assessment can be accompanied with an overestimation when enthalpy was used. Since local barometric pressure is another contributor factor for computation of enthalpy, it seems that determination of one cut- off point for enthalpy can introduce some errors in assessment. For example, cite 9 had minimum height from see level compared to other sites in this study, and hence maximum barometric pressure. So it may be necessary considering other meteorological variables inherent to different regions, such as, for example, atmospheric pressure^13^.

In high humid and very warm to hot regions, for example in regions 9 and 6, it seems that the air temperature has been a more influential factor than relative humidity. So, it can be expected that as shown in Table 4, assessment based on enthalpy probably be associate with an overestimation compared to when assessment done using WBGT. Therefore, in spite of good correlation can be observed between two indices (Figure 2-i), there is also a contradiction finding when categorization of indices was made (Table 4).

**Table 4:** Number of agreement and disagreement cases observed between WBGT and enthalpy

**Enthalpy (KJ.Kg** ^-1^ **)**		**WBGT (°C)**		**Kappa value**
		**Permissible**	**Impermissible**	
Site 1 (n=150)	Acceptable	125	11	0.680
	Unacceptable	0	14	
Site 2 (n=162)	Acceptable	76	0	0.853
	Unacceptable	12	74	
Site 3 (n=180)	Acceptable	129	0	0.870
	Unacceptable	9	42	
Site 4 (n=180)	Acceptable	172	0	a
	Unacceptable	8	0	
Site 5 (n=144)	Acceptable	59	0	0.860
	Unacceptable	10	75	
Site 6 (n=162)	Acceptable	0	0	b
	Unacceptable	0	162	
Site 7 (n=144)	Acceptable	91	0	0.924
	Unacceptable	5	48	
Site 8 (n=150)	Acceptable	83	0	0.891
	Unacceptable	8	59	
Site 9 (n=180)	Acceptable	91	0	0.272
	Unacceptable	65	24	
Total (n=1452)	Acceptable	826	11	0.815
	Unacceptable	117	498	

^a^ No statistics are computed because WBGT is a constant. ^b^ No statistics are computed because enthalpy and WBGT are constants.

Anyway, considering some restrictions such as inappropriateness of the enthalpy index for areas with very hot or humid conditions (restrictions seen in the case of WBGT index as a standard heat stress index), enthalpy can be introduced as a heat stress index just for preliminary assessments. Researchers working with thermal comfort have been using enthalpy to measure thermal energy inside rural facilities, establishing indicator values for many situations of thermal comfort and heat stress^13^. Recently, the "Heat Strain Score Index", HSSI, which is a questionnaire-based approach, was also introduced for a preliminary assessment of heat strain in warm climate conditions of Iran, but not necessarily generalizable to other climate conditions in Iran^31^.

Of course, preliminary assessment based on enthalpy indicator can be very helpful when, for example, reported by a meteorological agency for governmental responders, public society, occupational outdoor workers, the elderly, pregnant women and so on, to make appropriate management decisions and take preventive measures in time.

## Conclusions

Because of simplicity in measurement, applicability of the indicator for weather agencies, the consistency observed between enthalpy and a valid and accurate index (WBGT), sensor requirements which take only a few seconds to reach equilibrium and so on, enthalpy indicator can be introduced and applied as a good substitute for WBGT for outdoor settings. The best advantage of the enthalpy compared with WBGT is the possibility of reporting and notifying the public such as the other environmental parameters reported daily by meteorological data by every synoptical station all around the world. Besides, in contrast to WBGT, it does not have any correction or estimation for calculation while it will be covering a wide range of personal and environmental parameters currently observed in outdoor environments. The results of this study can be used not only in Iran, but also can be applied to all regions around the world with the same weather conditions. However, it is strongly recommended that the criterion for the selection of enthalpy in this study must be examined against other heat stress indices especially heat related physiological responses such as core temperature. As well as, since it is better for strength of an index that to be capable to define several ranges (more than two thermal regions, as the same of what defined in the present study) regarding heat stress intensity for subjects who exposed to interested environmental condition, more investigations are recommended to examine main heat related physiological or subjective responses against the new enthalpy indicator (either in field or experimental study).

## Acknowledgments

This research has been supported by Tehran University of Medical Sciences and Institute for Environmental Research (GrantNo.92-02-27-22837). We would like also to thank the Environmental and Occupational Health Center (EOHC) for providing the required data.

## Conflict of interest statement

The authors declare that there is no conflict of interest.

## Highlights

- Air enthalpy can integrate the effects of air temperature, humidity and air pressure
- A good consistency between enthalpy and WBGT as a standard heat index was determined
- Enthalpy Indicator can be used as an alternative to WBGT for heat stress assessment
- The cut-off point equal to 74.24 KJ.Kg-1 was determined as a criterion for enthalpy
- The ability to use meteorological data is one of the strengths of the enthalpy index
